# Evidence for the Presence of Non-Celiac Gluten Sensitivity in Patients with Functional Gastrointestinal Symptoms: Results from a Multicenter Randomized Double-Blind Placebo-Controlled Gluten Challenge

**DOI:** 10.3390/nu8020084

**Published:** 2016-02-08

**Authors:** Luca Elli, Carolina Tomba, Federica Branchi, Leda Roncoroni, Vincenza Lombardo, Maria Teresa Bardella, Francesca Ferretti, Dario Conte, Flavio Valiante, Lucia Fini, Edoardo Forti, Renato Cannizzaro, Stefania Maiero, Claudio Londoni, Adriano Lauri, Giovanni Fornaciari, Nicoletta Lenoci, Rocco Spagnuolo, Guido Basilisco, Francesco Somalvico, Bruno Borgatta, Gioacchino Leandro, Sergio Segato, Donatella Barisani, Gaetano Morreale, Elisabetta Buscarini

**Affiliations:** 1 Center for the Prevention and Diagnosis of Celiac Disease, Gastroenterology and Endoscopy Unit, Fondazione IRCCS Ca’ Granda Ospedale Maggiore Policlinico, via Francesco Sforza 35, 20122 Milano, Italy; tomba.carolina@gmail.com (C.T.); federica.branchi@gmail.com (F.B.); leda.roncoroni@tiscali.it (L.R.); vicky.l@hotmail.com (V.L.); mariateresa.bardella@yahoo.com (M.T.B.); francesca.ferretti01@gmail.com (F.F.); dario.conte@unimi.it (D.C.); 2 Department of Medical, Surgical and Transplant Physiopathology, University of Milan, via Francesco Sforza 35, 20122 Milan, Italy; 3 Department of Biomedical, Surgical and Dental Sciences, University of Milan, via Festa del Perdono 7, 20122 Milan, Italy; 4 Gastroenterology and Digestive Endoscopy Unit, Santa Maria del Prato Hospital, via Bagnols sur Ceze 1, 32032 Feltre, Italy; flavio.valiante@gmail.com; 5 Department of Internal Medicine, Gastroenterology and Digestive Endoscopy Unit, Busto Arsizio Hospital, via A. Da Brescia 1, 21052 Busto Arsizio, Italy; finilucia@yahoo.it; 6 Operative Endoscopy Unit, Niguarda Ca’ Granda Hospital, Piazza Ospedale Maggiore 3, 20162 Milan, Italy; edoardo.forti@ospedaleniguarda.it; 7 Gastroenterology, CRO National Cancer Institute, via Franco Gallini 2, 33081 Aviano, Italy; rcannizzaro@cro.it (R.C.); smaiero@cro.it (S.M.); 8 Gastroenterology and Endoscopy Unit, Crema Maggiore Hospital, Largo Dossena 2, 26013 Crema, Italy; c.londoni@hcrema.it (C.L.); ebuscarini@rim.it (E.B.); 9 Gastroenterology Unit, Ospedale Civile Santo Spirito, via Fonte Romana 8, 65100 Pescara, Italy; adriano.lauri@tin.it; 10 Medicine and Gastroenterology Unit, IRCCS Arcispedale S. Maria Nuova, via Risorgimento 80, 42100 Reggio Emilia, Italy; Giovanni.Fornaciari@asmn.re.it; 11 Gastroenterology Unit, Ospedale Valduce, via Dante Alighieri 11, 22100 Como, Italy; nicolettalenoci@libero.it; 12 Department of Experimental and Clinical Medicine, Magna Græcia University, Loc. Germaneto, 88100 Catanzaro, Italy; rocco.spagnuolo79@gmail.com; 13 Gastroenterology and Endoscopy Unit, Fondazione IRCCS Ca’ Granda Ospedale Maggiore Policlinico, via Francesco Sforza 35, 20122 Milano, Italy; basilisc@policlinico.mi.it; 14 Alphasearch, via Gianfrancesco Parravicini 40, 20900 Monza, Italy; fsomlav@tin.it; 15 Medicine Unit, Ospedale San Biagio, Piazza Vittime dei Lager Nazifascisti 1, 28845 Domodossola, Italy; borgattabruno@alice.it; 16 Gastroenterological Department, IRCCS “De Bellis” Hospital, SP Turi 27, 70013 Castellana Grotte (BA), Italy; leandro@media.it; 17 Gastroenterology and GI Endoscopy Unit, Macchi Hospital-Varese, Viale Luigi Borri 57, 21100 Varese, Italy; sergio.segato@katamail.com; 18 School of Medicine and Surgery, University of Milano-Bicocca, via Cadore 48, 20900 Monza, Italy; donatella.barisani@unimib.it; 19 Gastroenterology Unit, Policlinico of Palermo, via del Vespro 129, 90127 Palermo, Italy; gaetanocmorreale@libero.it

**Keywords:** non-celiac gluten sensitivity, gluten-free diet, double-blind placebo controlled challenge, functional gastrointestinal disorders, irritable bowel syndrome

## Abstract

Non-celiac gluten sensitivity (NCGS) is characterized by the onset of symptoms after eating gluten-containing food. We aimed to single out NCGS subjects among subjects with functional gastrointestinal symptoms. Patients were enrolled in a multicenter double-blind placebo-controlled trial with crossover. Symptoms and quality of life were evaluated by means of 10-cm VAS and SF36. Iron parameters, transaminases and C reactive protein (CRP) were evaluated. After a three-week-long gluten-free diet (GFD), responsive patients were randomly assigned to gluten intake (5.6 g/day) or placebo for seven days, followed by crossover. The primary endpoint was the worsening of symptoms (VAS increase ≥3 cm) during gluten ingestion compared to placebo. One hundred and forty patients were enrolled and 134 (17 males, mean age 39.1 ± 11.7 years, BMI 22.4 ± 3.8) completed the first period. A total of 101 subjects (10 males, mean age 39.3 ± 11.0 years, BMI 22.3 ± 4.0) reported a symptomatic improvement (VAS score 2.3 ± 1.2 *vs.* 6.5 ± 2.2 before and after GFD, *p* = 0.001). 98 patients underwent the gluten challenge and 28 (all females, mean age 38.9 ± 12.7 years, BMI 22.0 ± 2.9) reported a symptomatic relapse and deterioration of quality of life. No parameters were found to be statistically associated with positivity to the challenge. However, 14 patients responded to the placebo ingestion. Taking into account this finding, about 14% of patients responding to gluten withdrawal showed a symptomatic relapse during the gluten challenge. This group is suspected to have NCGS.

## 1. Introduction

Non-celiac gluten sensitivity (NCGS) is a “syndrome characterized by intestinal and extra-intestinal symptoms related to the ingestion of gluten-containing food, in subjects that are not affected by either celiac disease (CD) or wheat allergy (WA)” [1,2,3]. Although reports of patients presenting gluten-responsive clinical pictures in absence of CD have been published since the 1970s [4,5], it was only in 2012 that a revision of the nomenclature for gluten-related disorders included NCGS [6].

The NCGS clinical picture is heterogeneous and not specific, including intestinal (diarrhea, constipation, bloating, nausea and epigastric pain) and extra-intestinal (lack of well-being, anxiety, tiredness, fibromyalgia, chronic fatigue, foggy mind and headache) symptoms [2]. The exclusion of CD or WA and a response to a gluten-free diet (GFD) are actually the main parameters used to identify this condition. Available blood tests and duodenal histology (usually unremarkable) do not help towards the differential diagnosis [6,7]. The placebo effect as well as the presence in the food ingested of other active molecules (amylase trypsin inhibitor, ATI) or fermentable substrates (fermentable oligo-di and mono-saccharides and polyols, FODMAPs) may act as important confounders [8,9,10]. With NCGS gaining wide interest, general practitioners and specialists face an increasing number of patients often embarking on self-administered GFD without any medical indications [11,12]. Consequently, a correct diagnosis is necessary to appropriately manage these patients and to avoid useless and costly diets. Moreover, if a large proportion of patients with undetermined gastrointestinal symptoms or irritable bowel syndrome (IBS) were dietetically treated, this would lead to a reduced need for drugs with direct and indirect economic advantages [9,13].

The current literature on NCGS consists of a limited number of studies and contains conflicting results mainly due to retrospective protocols [14], limited sample size [15,16], and single center design [13,14,16,17,18]. Of these studies, none followed the recently suggested steps (the Salerno Experts’ criteria) for NCGS diagnosis, starting with the evaluation of the GFD effect in selecting patients suitable for a gluten challenge [7].

In particular, there is still a need of a multicenter double-blind placebo-controlled gluten challenge (considered the most powerful diagnostic weapon in this scenario), which helps to single out NCGS patients in the “real-life” setting of gastroenterological services.

Thus, the aim of our study was to identify patients with NCGS from those reporting an improvement of gastrointestinal symptoms after GFD through a double-blind placebo-controlled gluten challenge with crossover.

## 2. Experimental Section

### 2.1. Study Design and Patients

The study was carried out in 15 gastroenterological out-patient centers in Italy. The enrolling centers were coordinated by the Center for the Prevention and Diagnosis of Celiac Disease—Gastroenterology and Endoscopy Unit of the Fondazione IRCCS Ca’ Granda Ospedale Maggiore Policlinico in Milan. The study was approved by the Ethics Committee of the Fondazione IRCCS Ca’ Granda (protocol number 453/14) and notified by all the Ethics Committees of the participating centers. The trial was registered in ClinicalTrial.gov (NCT01864993) with the acronym “GLUTOX”. A dedicated webpage [19] and YouTube channel [20] were created.

The trial was supported by the Italian Society of Hospital Gastroenterologists and Endoscopists (AIGO).

Between 2 September 2013 and 18 November 2014, patients aged 18–75 years, giving their written informed consent to participate in the study, were recruited from the gastroenterological out-patient centers. The inclusion criteria were in accordance with the recent NCGS consensus [1]: The study enrolled 140 adults (age ≥18 years), routinely attending the gastroenterological outpatient clinic. They reported functional gastroenterological symptoms according to the Rome III criteria [21], (40 IBS with diarrhea, 14 IBS with constipation, 20 mixed IBS, three unsubtyped IBS, 12 dyspeptic with postprandial distress syndrome, 10 dyspeptic with epigastric pain syndrome and 41 presenting other functional gastrointestinal symptoms) following a gluten-containing diet and with negative anti tissue transglutaminase IgA, normal IgA dosage negative IgE mediated WA verified by means of a skin prick test and serological IgE dosages. In case of high CD suspicion (for example in subjects with a first-degree relative with CD), a duodenal biopsy was performed for the identification of seronegative patients according to the guidelines issued by the Italian Ministry of Health [22] and the international recommendations [23]. CD testing was performed during a gluten-containing diet. The exclusion criteria were: CD, WA, inflammatory bowel diseases, psychiatric disorders, major abdominal surgery (in particular intestinal resections), diabetes mellitus, systemic autoimmune diseases, previous anaphylactic episodes, any systemic disorders, patients already following or having followed a GFD regimen in the previous six months, pregnant or breast-feeding women, and patients already on pharmacological therapy.

Meetings and conference calls were organized before and during the trial to standardize the evaluation criteria.

### 2.2. Protocol

The trial was articulated in two consecutive phases. Phase 1 investigated the subjects’ responses to GFD; Phase 2 included the patients reporting a symptomatic benefit from GFD (*i.e.*, GFD responsive) who were randomized for the double-blind gluten challenge.

*Phase 1*. At enrollment patients were following a gluten-containing diet; the Rome III criteria and the demographic parameters were recorded. Patients were asked to fill in a questionnaire about their perceived level of physical and mental health (SF36 questionnaire) and a series of 10-cm long visual analogue scales (VASs) referring to the level of satisfaction about their health status and the severity of specific symptoms (abdominal pain, satisfaction with stool consistency, bloating, postprandial fullness, early satiety, epigastric pain and other symptoms). A further VAS evaluated the satisfaction about the general well-being; only those subjects reporting a satisfaction ≤4 cm were enrolled (0 being extremely poor satisfaction and 10 very high satisfaction). After this initial evaluation, the patients were asked to follow a strict 3-week-long GFD. The GFD regimen was illustrated by dedicated medical personnel. Patients were instructed about the GFD and provided with flyers describing it, listing allowed and not allowed foods and advising on the way to read the food labels. The information had been developed by an expert nutritionist (LR). The enrolled patients were also given direct contact links (by e-mail and telephone) to their enrollment centers for any query about their diet.

At the end of Phase 1, the patients were asked to fill in the VASs and SF36 questionnaire. Only those patients presenting a significantly improvement (baseline ΔVAS ≥ 3 cm) in the general well-being VAS were defined as “GFD responsive” and carried on to Phase 2. The patients with no improvement of their general well-being VAS were considered “non-responsive” and terminated the trial.

*Phase 2*. The GFD-responsive patients were invited to maintain a strict GFD and underwent a placebo-controlled double-blind gluten challenge with crossover. The patients were randomized to take gluten or placebo for 7 days. The daily amount of gluten administered (Uniglad Ingredienti S.r.l., Cuneo, Italy) was 5.6 g equivalent to the gluten content of an 80 g pasta portion. The gluten used contained 83% protein; the non-protein part was mainly made of starch (15%) and ash (<1%). The gluten was further analyzed, resuspended in Laemmli buffer, and analyzed by 10% SDS-PAGE to evaluate its components. After separation, proteins were stained for 30 min with Coomassie Brilliant Blue R-250, and then gels were de-stained with methanol/water/acetic acid (50/40/10, v/v/v) overnight. The identified bands had molecular weights comparable to those reported for high or low-molecular-weight glutenins, as well as gliadins. An amount of non-gluten proteins with a molecular weight below 17 κDa was found.

Gluten or placebo were administered as 7 gastrosoluble capsules (0.8 g of gluten per capsule) per day (4 at lunch and 3 at dinner). Rice starch was chosen as placebo because of its low fermentable capability consequent to rapid absorption [24]. A 7-day-long wash-out was scheduled between the 7-day sequences of capsules (placebo/gluten or gluten/placebo). The total duration of Phase 2 was 21 days (always on a GFD): 7 days on gluten or placebo capsules, 7 days wash-out and 7 days on placebo or gluten depending on randomization. At the end of each sequence the patients were asked to complete the symptom VASs and SF36 questionnaire. The patients who reported the worsening of their general well-being, *i.e.*, ΔVAS ≥ 3 cm, while taking gluten capsules compared to the placebo, were considered sensitive to gluten.

### 2.3. Randomization and Masking

The patients who were GFD responsive in Phase 1 were randomly allocated—according to a computer-generated series—to take gluten or placebo-containing capsules as first treatment. The capsules containing gluten or placebo were completely undistinguishable and were administered to the patients via white anonymous sealed plastic boxes. Each box was only marked with a serial number assigned by the independent laboratory producing the capsules (Moldes S.r.l., Corsico, Italy). Only the independent company specialized in biostatistics (Alphasearch, Monza, Italy) was in the position to associate the capsule box numbers with their content at the protocol completion. The capsule supplier and the biostatistics company were not otherwise connected to the study and all the personnel managing patients had no access to the allocation sequence until the end of the protocol.

### 2.4. Statistical Analysis

A per-protocol analysis was applied to the trial. All the enrolled patients data were registered in a central database (FileMaker Pro software ver. 12, Santa Clara, CA, USA) managed by an independent statistical company (Alphasearch, Monza, Italy). Hypotheses were verified using SPSS ver. 18 (IBM SPSS, Milano, Italy) and a *p* value < 0.05 was considered as statistically significant (test significance level: 5%, two tails); GraphPad Prism ver. 5 (GraphPad Software, San Diego, CA, USA) was used to draw graphs. Kolmogorov-Smirnov’s test was used to assess the normal distribution of data. Data were described as mean and standard deviation (SD). Continuous variables were analysed by *t* or Mann-Whitney tests. Categorical variables were compared by χ^2^ or Fisher’s exact tests. During Phase 1 VAS values were compared via *t* test for paired samples. The One-sample *t* test and Wilcoxon Signed-Rank Test were used for one-column statistics. SF36 parameters were standardized [25], grouped and compared via *t* test for paired samples. The between-within groups study was conducted via ANOVA variance analysis. Tukey’s method for multiple comparisons was used as the confirmatory test for both VAS and SF36. Assuming a 15% positivity to the challenge, we estimated that 90 patients would be needed to achieve a power of 80% (β-1) and a 2-sided 5% significance level [26] (G*Power package ver. 3.1.9.2, University of Dusseldorf [27]).

### 2.5. Funding Sources

The study was independent and endorsed by AIGO (Italian Society of Hospital Gastroenterologists and Endoscopists). Logistic costs were covered by an unconditioned grant from the Dr. Schär Institute. The remaining costs were met directly by the coordinating center. Only the research team members had access to the study data and their interpretation; they reviewed and approved the final manuscript.

## 3. Results

### 3.1. Phase 1: Outcome of Patients Following the Gluten Free Diet

**Figure 1:**
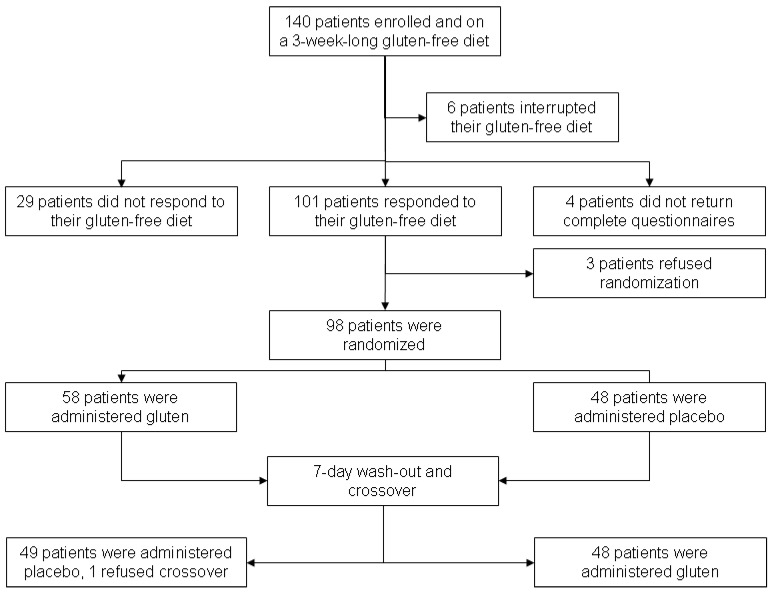
Trial profile.

According to the enrollment criteria, 140 patients were included in the study but six interrupted their GFD regimen because of social commitments clashing with their GFD requirements. In total, 134 patients correctly completed the 3-week GFD course but four patients incorrectly filled in the case report forms and thus were excluded from the later evaluation of responsiveness *vs.* non-responsiveness to GFD. Figure 1 and Table 1 show the trial profile and the clinical and demographic characteristics of the patients. Following the criteria of defining GFD responsiveness (improvement of the global well-being VAS ≥ 3 cm), 101 patients (75.3%) were found to be responsive. Table 2 provides the VAS values at the beginning of the trial and at the end of Phase 1 (after three weeks on GFD). The overall values and scores obtained by GFD-responsive *vs.* non-responsive patients are reported, including the between-within groups’ analysis. The improvement of VAS scores was associated with an increase of the quality of life (QoL) as shown by the SF36 physical and mental summary components. The SF36 overall physical and mental summary components at enrollment and after the 3-week long GFD regimen were 42.7 ± 8.0 *vs.* 48.9 ± 6.3 (*p* < 0.001) and 42.6 ± 8.3 *vs.* 48.4 ± 7.3 (*p* < 0.001), with an improvement of 14.5% and 13.8%, respectively. In the GFD-responsive group, the physical and mental summary components passed from 43.6 ± 8.7 to 50.9 ± 6.3 (*p* < 0.001) and from 41.9 ± 9.3 to 48.5 ± 8.4 (*p* < 0.001) at enrollment and after GFD, respectively. Conversely, in the GFD non-responsive group, the physical and mental summary components did not change, with values from 44.8 ± 8.3 to 45.7 ± 6.7 (*p* = 0.92) and 41.9 ± 10.3 and 43.6 ± 9.5 (*p* = 0.70) at enrollment and after GFD, respectively.

No adverse events were registered during the 3-week GFD course.

**Table 1:** Clinical and demographic characteristics of enrolled patients during Phase 1 of the GLUTOX trial (see Methods section). The characteristics of all the patients, distinguishing those responsive/non-responsive to the gluten-free diet (GFD), are reported.

Variable	Overall (*n* = 134 *)	GFD		*p*
		Responsive (*n* = 101)	Non-Responsive (*n* = 29)	
Sex				
Male	17 (12.7%)	10 (9.9%)	7 (24.1%)	0.06
Female	117 (87.3%)	91 (90.1%)	22 (75.9%)	
Age: years	39.1 (11.7)	39.3 (11.0)	38.5 (13.6)	0.75
BMI	22.4 (3.8)	22.3 (4.0)	22.4 (3.2)	0.96
Functional disease				
IBS	77 (57.5%)	55 (54.5%)	20 (69.0%)	0.80
Dyspepsia	22 (16.4%)	18 (17.8%)	3 (10.3%)	
Other	35 (26.1%)	28 (17.7%)	6 (20.7%)	
IDA	27 (20.1%)	21 (20.8%)	6 (20.6%)	0.47
AST increased	0 (0%)	0 (0%)	0 (0%)	-
ALT increased	3 (2.2%)	3 (2.9%)	0 (0%)	0.61
CRP increased	4 (2.9%)	3 (2.9%)	1 (3.4%)	0.54
Presence of a first-degree relative with CD	16 (11.9%)	13 (12.9%)	1 (3.4%)	0.19

ALT, Alanine Transaminase; AST, Aspartate Transaminase; BMI, Body Mass Index; CD, Celiac Disease; CRP, C Reactive Protein; GFD, Gluten-Free Diet; IDA, Iron Deficiency Anemia; IBS, Irritable Bowel Syndrome; * Four with incomplete questionnaire (see also Figure 1).

**Table 2:** Visual analogue scale (VAS) values of symptoms in patients responsive and non-responsive to the gluten-free diet (GFD) and in the overall series of patients. Statistical analysis was performed to evaluate the significance of the comparisons before and after the gluten-free diet (P_(0–21)_) and between the responsive and non-responsive groups (P_(R *vs.* NR)_) at the end of the gluten-free diet.

Variable	VAS Values		*p* (0–21)	*p* (R *vs.* NR)
	At Enrollment	After 3 Weeks on GFD		
Abdominal pain				
Overall	5.9 ± 2.8	2.7 ± 2.6	0.001	0.001
GFD responsive	6.1 ± 2.7	2.0 ± 2.1	0.001	
GFD non-responsive	5.2 ± 3.0	5.0 ± 2.6	0.66	
Stool Consistency satisfaction				
Overall	3.5 ± 2.7	6.3 ± 2.7	0.001	0.001
GFD responsive	3.6 ± 2.7	7.1 ± 2.2	0.001	
GFD non-responsive	3.3 ± 2.6	3.7 ± 2.6	0.47	
Bloating				
Overall	6.8 ± 2.7	3.4 ± 2.9	0.001	0.001
GFD responsive	7.1 ± 2.4	2.7 ± 2.5	0.001	
GFD non-responsive	6.0 ± 3.3	5.8 ± 3.0	0.80	
Postprandial fullness				
Overall	7.2 ± 2.4	3.9 ± 2.5	0.001	0.580
GFD responsive	7.6 ± 2.1	3.8 ± 2.4	0.001	
GFD non-responsive	5.2 ± 3.4	4.1 ± 3.5	0.47	
Early Satiety				
Overall	5.7 ± 3.3	2.4 ± 2.4	0.001	0.770
GFD responsive	6.2 ± 3.1	2.5 ± 2.5	0.001	
GFD non-responsive	3.0 ± 3.3	1.6 ± 1.5	0.32	
Epigastric pain				
Overall	6.1 ± 2.9	2.9 ± 2.9	0.001	0.470
GFD responsive	6.4 ± 2.8	2.8 ± 2.9	0.001	
GFD non-responsive	4.7 ± 3.0	3.2 ± 3.1	0.06	
Other symptoms				
Overall	7.3 ± 2.4	3.8 ± 3.5	0.005	0.100
GFD responsive	7.5 ± 1.9	3.1 ± 2.9	0.001	
GFD non-responsive	6.2 ± 4.5	7.0 ± 5.1	0.87	
Global satisfaction				
Overall	2.3 ± 1.2	6.5 ± 2.2	0.0001	0.001
GFD responsive	2.2 ± 1.0	7.4 ± 1.4	0.0001	
GFD non-responsive	2.5 ± 1.3	3.4 ± 1.5	0.001	

GFD, gluten-free diet; VAS, Visual Analogic Scale; R and NR, responsive and non-responsive; 0-21, before and after gluten-free diet.

### 3.2. Phase 2: Outcome of the Gluten Challenge

**Figure 2:**
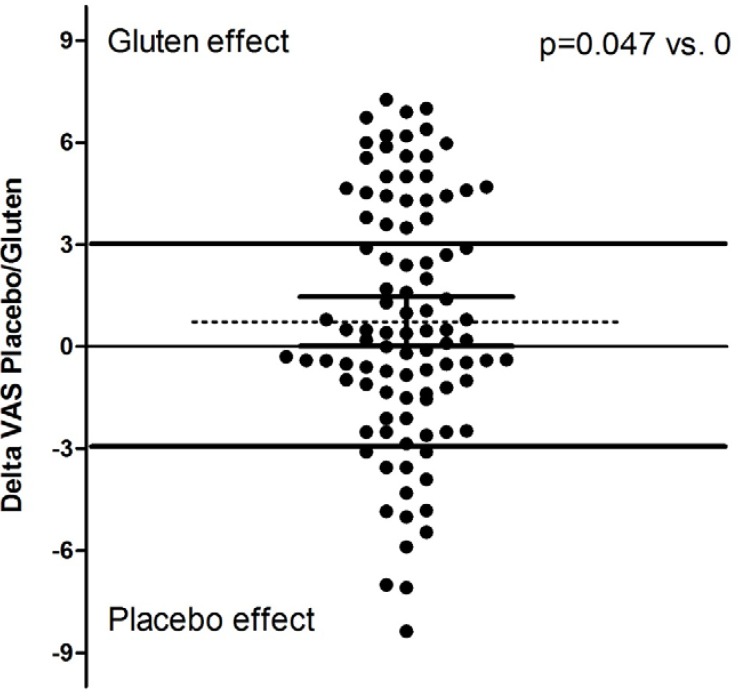
Distribution of the differences between the global well-being visual analogue scale (VAS) after the gluten *vs.* placebo challenge. The dotted line shows the mean (*p* = 0.047 *vs.* 0). 95% CI are reported (0.01–1.5). The continuous lines show the identity line (=0), the cut-off of the difference (3) chosen to define patients with NCGS and the line identifying patients of the placebo responsive group (≤−3).

Among the 101 GFD-responsive patients, 98 underwent the double-blind placebo-controlled challenge (DBPCC) with gluten (three refused the challenge fearing a relapse of symptoms during it). On maintaining a GFD, 50 subjects took gluten at first and, among them, one patient interrupted the trial (see Figure 1 for details). Overall, the subjects reported a greater deterioration of their well-being during gluten than during placebo administration (5.3 ± 2.5 *vs.* 6.1 ± 2.4, *p* = 0.05). Figure 2 shows the distribution of the differences (Δ) between global VAS values after gluten and placebo treatment. In particular, the mean ΔVAS value was 0.74 ± 3.62, median 0.40 (95% CI 0.01–1.47), *p* = 0.047 *vs.* “0” value (*i.e.*, same effect of gluten and placebo); a positive ΔVAS pushed towards a gluten effect. By grouping the randomized subjects as “positive” (with a symptomatic relapse during blind gluten ingestion) and “negative” (without any symptomatic reaction during gluten administration), 28 patients were found to be positive and 69 negative. Table 3 provides the clinical and demographic parameters of the randomized subjects: overall, positive to DBPCC and negative to DBPCC. No demographic, clinical or biochemical factors (iron parameters, transaminases and CRP) were found to be associated with the gluten challenge response. Evaluating the other symptomatic VAS values, some were revealed to be associated to the blind gluten challenge in patients grouped “positive” to DBPCC (Table 4). In line with the VAS changes, both mental and physical components of SF36 were significantly lower in patients positive to the challenge than in those (Table 5).

Among the positive patients, 15 (53%) took gluten as the first treatment without any statistical effect of the capsule sequence. Moreover, the overall VAS values during the first and the second treatment did not show a significant difference thus excluding a carry-over effect (5.6 ± 2.4 and 5.9 ± 2.7, respectively). On applying the criteria used to define gluten responsiveness, 14 patients could be considered placebo responsive, indicating a possible nocebo effect. Notably, the placebo-responsive group is composed of half of the gluten-responsive patients (14 *vs.* 28, *p* < 0.05). During the first week after randomization, 74% and 73% of the subjects taking gluten and placebo, respectively, completed all the 49-capsule course without any statistical difference (the mean number of gluten and placebo capsules per subject 44.9 ± 8.9 and 44.6 ± 9.6, respectively, *p* = 0.78). Similar results were obtained during the second treatment following the wash-out interval. Only one mild adverse event (a mild periorbital edema) was recorded during the placebo administration.

**Table 3:** Clinical and demographic characteristics of randomized patients during Phase 2 of the GLUTOX trial. The characteristics of the entire group of patients and those positive/negative to the gluten challenge are reported.

Variable	Overall (*n* = 98)	DBPCC Positive (*n* = 28)	DBPCC Negative (*n* = 69)	*p*
Sex				
Male	10 (10.2%)	0 (0%)	10 (14.5%)	0.08
Female	88 (89.8%)	28 (100%)	59 (85.5%)	
Age years	39.4 ± 11.1	39.9 ± 12.7	39.2 ± 10.6	0.79
BMI	22.4 ± 4.1	22.0 ± 2.9	22.6 ± 4.5	0.51
Functional disease				
IBS	53 (54.1%)	18 (64.3%)	35 (50.7%)	0.26
Dyspepsia	17 (17.3%)	4 (14.3%)	13 (18.8%)	
Other	28 (28.6%)	6 (21.4%)	21 (30.5%)	
IDA	10 (10.2%)	4 (14.2%)	3 (4.3%)	0.47
AST increased	0 (0%)	0 (0%)	0 (0%)	
ALT increased	3 (3.0%)	0 (0%)	0 (0%)	0.61
CRP increased	1 (1.0%)	0 (0%)	1 (1.4%)	0.54
Presence of a first-degree relative with CD	14 (14.3%)	5 (21.4%)	9 (3.4%)	0.19

ALT, Alanine Transaminase; AST, Aspartate Transaminase; BMI, Body Mass Index; CD, Celiac Disease; CRP, C-Reactive Protein; DBPCC, Double-Blind Placebo-Controlled Challenge; GFD, Gluten-Free Diet; IDA, Iron Deficiency Anemia; IBS, Irritable Bowel Syndrome. One patient dropped out during the challenge.

**Table 4:** Visual analogue scale (VAS) values of patients according to their positive/negative response to the gluten challenge.

Variable	VAS Values		*p*
	DBPCC Positive	DBPCC Negative	
Abdominal pain	5.4 ± 2.4	3.2 ± 2.8	0.006
Stool consistency satisfaction	4.5 ± 2.9	5.7 ± 3.0	0.08
Bloating	8.2 ± 2.8	3.6 ± 2.9	0.0001
Postprandial Fullness	6.6 ± 3.0	4.9 ± 2.9	0.01
Early satiety	6.4 ± 2.8	4.4 ± 2.9	0.03
Epigastric pain	2.3 ± 2.3	3.0 ± 3.0	0.27
Other gastrointestinal symptoms	4.6 ± 4.0	3.4 ± 3.0	0.41

DBPCC, double-blind placebo-controlled challenge.

**Table 5:** SF36 scores of patients positive and negative to the gluten challenge. Noteworthy, in patients positive to the gluten challenge, a deterioration of satisfaction after gluten assumption is associated with a significantly reduced SF36 score.

Variable	SF36 Values		*p*
	DBPCC Positive (*n* = 28)	DBPCC Negative (*n* = 69)	
Physical Functioning	46.9 ± 11.6	52.3 ± 6.8	0.030
Role Limitation—Physical	42.6 ± 11.6	49.6 ± 9.7	0.003
Bodily Pain	46.9 ± 9.9	41.0 ± 10.5	0.010
General Medical Health	44.0 ± 9.2	45.6 ± 10.2	0.480
Vitality	44.2 ± 9.2	50.7 ± 8.5	0.001
Social Functioning	41.6 ± 11.2	47.9 ± 9.6	0.007
Role Limitation Emotional	42.6 ± 11.9)	50.5 ± 9.4	0.003
Mental Health	44.7 ± 8.0	48.4 ± 8.5	0.050
Physical Summary Component	44.4 ± 9.2	49.1 ± 7.6	0.010
Mental Summary Component	43.0 ± 8.8	48.9 ± 8.6	0.004

DBPCC, Double-Blind Placebo-Controlled Challenge.

## 4. Discussion

Our study demonstrated that, among the larger cohort of patients responsive to GFD, about 14% showed a symptomatic relapse during the blind placebo-controlled gluten challenge and, accordingly, they can be defined as patients with NCGS, confirming that gluten ingestion may induce gastrointestinal symptoms and impairment of quality of life. This study could be considered the first attempt to assess the performance of the Salerno Experts’ diagnostic criteria for NCGS in a daily clinical setting [7].

NCGS is a syndrome characterized by intestinal and extra-intestinal symptoms induced by the ingestion of gluten-containing food, once CD and WA have been excluded [2]. This definition has raised some skepticism among the scientific community [28,29] because determining the “functional” nature of these patients is usually affected by a strong placebo effect [8]. Consequently, in the absence of reliable biomarkers, the introduction of a gluten challenge structured as a double-blind placebo-controlled trial with crossover was considered necessary to diagnose and stratify these patients, as recently underlined by the Salerno Experts’ Criteria [7,23].

The results of our study suggest that gluten can be a major trigger for gastrointestinal symptoms in line with previous reports [13,15,17]. In contrast, other authors would prefer to replace the definition “NCGS” with “non-celiac wheat sensitivity” (NCWS) underlining the fact that, in addition to gluten, other potentially bioactive components of wheat and related cereals (e.g., ATIs and FODMAPs) are also excluded during GFD [18,30,31]. Also in our study, the presence of non-gluten proteins in any amount (especially ATI) could play a role, as shown by SDS-PAGE analysis.

The current literature is conflicting on this matter. Biesiekierski *et al.* [17] firstly described the symptomatic effect following the blinded intake of gluten in a group of IBS subjects, reporting a rapid (within two days) symptomatic onset. However, in a successive trial, the same research team found no gluten effect in patients with self-reported NCGS [18]. Successively, Carroccio *et al.* [14] demonstrated that about 30% of functional gastrointestinal patients responded to a wheat challenge and the authors defined them as NCWS patients. More recently, Di Sabatino *et al.* [15] challenged 4.375 g of gluten in a double-blind trial with crossover and evidenced a gluten response in 20% of their patients, in line with the results from Shahbazkhani *et al.* [13]. However, Zanini *et al.* [16] failed to evidence a relevant gluten effect in patients previously diagnosed as NCGS. Although conducted with different protocols, these data suggest a relevant effect of diet on functional gastrointestinal symptoms.

Different factors can influence the findings of the aforementioned trials. The gluten vehicle is crucial to maintaining blinding; in some studies, muffins [17], predefined diets [16,18] or powders [13,16] were used. In our study, capsules were used, with an extremely small possibility for doctors and patients to distinguish the capsules containing gluten (without opening capsules and testing the contained powders). However, a weak point of using capsules is their unnatural food format, not reflecting common eating and cooking practices in gluten ingestion.

Moreover, enrollment criteria and evaluation of GFD responses are crucial as underlined by the Salerno Experts’ Criteria [7]. Previous studies principally enrolled patients reporting a symptomatic benefit from GFD without any assessment of GFD or evaluation of pre-/post-GFD severity of symptoms. In such a composed cohort of patients, the placebo effect might well be strong.

In the absence of a dedicated and validated scoring system, we measured (by 10-cm long VAS) the global well-being level as the primary endpoint. The correctness of this approach is supported by the concordance with SF36 results, underlying the importance of adopting a patient-oriented outcome in assessing diet efficacy.

The correct timing of the gluten challenge and duration of the GFD course to evaluate responsiveness are both uncertain. Gluten symptoms usually arise and disappear quickly; for this reason, we chose to challenge gluten for a week, assuming this timeframe sufficient to define a clinical onset, maintaining a real-life scenario and avoiding drop-outs. Also, the dosage of gluten used in a challenge is not standardized and has been different in all the discussed studies; in our protocol 5.6 g of gluten per day was administered, roughly the amount of gluten contained in an 80 g pasta portion.

The main strength of our study rests on the blinding of patients and doctors, and the crossover design, which allows a patient-by-patient assessment. On the other hand, some weakness arises from the arbitrary choice of timing and gluten dosage; in fact, patients slow to respond or with a high response threshold might not be recognized. Moreover, the protocol did not make use of a scheduled diet besides GFD that was verified by the nutritionist at the moment of the planned visits; however, given the short timeframe of the gluten/placebo challenge, other diet variables cannot be excluded, including the presence of small amounts of ATI. In our protocol, CD was excluded following the international guidelines, which do not include duodenal biopsy in all cases but high-risk subjects only; on choosing this “real-life” CD screening, an extremely low probability to encounter seronegative CD patients inside the cohort must be accepted. Another weak point is the presence of other proteins beyond gliadins and glutenins in the gluten content; thus, the influence of other factors or cofactors cannot be excluded. In our study, symptomatic deterioration was also observed after placebo in a small but not negligible number of patients (14 placebo responders *vs.* 28 gluten responders), raising the possibility that at least in some patients with NCGS the response to the gluten challenge was only owing to chance. This situation can raise some doubts about the actual impact of NCGS in the cohort of investigated patients. On the other hand, it should also be recognized that some of the patients with a symptomatic deterioration of >0 but less than the arbitrary cut-off of 3 after the gluten challenge might also be affected by NCGS. Another factor suggesting a real gluten effect is the general deterioration of the VAS after blinded gluten administration (see Figure 2). However, following a conservative profile and considering half of patients responsive to gluten as “false” responders (taking into account the percentage of placebo responders), about 14% can be considered NCGS.

A relevant response rate (75%) after GFD was found at the end of the Phase 1 and noted. The presence of patients responsive to GFD but negative to the gluten challenge is intriguing. A relevant part of the symptomatic response to GFD may be justified by a placebo effect but some of such patients might be sensitive to other unspecified wheat components (ATI), additives or FODMAPs as previously discussed.

Although planned before the publication of the Salerno Experts’ Criteria, our discussed protocol presents numerous similarities and can be considered the first attempt to apply the Salerno Experts’ Criteria to the daily clinical practice. Firstly, the evaluation of GFD responsiveness and specific timeframes have been adopted, such as the use of VAS and cut-off values. Differently, capsules were used instead of bars and the number of observations was lower to maintain a realistic scenario, compatible with the daily clinical practice, which usually discourages time-consuming procedures. The Salerno Experts’ Criteria referred to the use of VAS to evaluate intestinal and extra-intestinal symptoms while we used a single VAS on well-being. However, the use of the quality of life questionnaire (SF36) can assist in the assessment of other-than-intestinal parameters.

## 5. Conclusions

Our protocol identified a smaller set of patients with NCGS among the group of GFD-responsive patients and this approach can be the starting point for developing a diagnostic tool for NCGS as indicated by the Salerno Experts’ Criteria. Moreover, the presented data have highlighted a decrease in the overall well-being and quality of life of patients with functional gastrointestinal symptoms while on a blinded gluten intake, confirming the induction of intestinal and extra-intestinal symptoms and that GFD can have a beneficial effect even in the absence of CD or WA. Identifying non-celiac gluten-sensitive patients through a gluten challenge as described in the “Glutox” trial allows us to target patients undergoing dietary restrictions.
